# Menopause and Metabolic Syndrome in Tunisian Women

**DOI:** 10.1155/2014/457131

**Published:** 2014-03-31

**Authors:** Samir Ben Ali, Hanen Belfki-Benali, Hajer Aounallah-Skhiri, Pierre Traissac, Bernard Maire, Francis Delpeuch, Noureddine Achour, Habiba Ben Romdhane

**Affiliations:** ^1^Laboratory of Epidemiology and Prevention of Cardiovascular Disease, Faculty of Medicine, 15 rue Djebel Akdhar, La Rabta, Bab Saâdoun, 1007 Tunis, Tunisia; ^2^Institut de Recherche pour le Développement (IRD), UMR 204 NUTRIPASS, IRD-UM1-UM2, 911 Avenue Agropolis, 34394 Montpellier, France

## Abstract

*Objectives*. This study aimed to evaluate the effect of menopausal status on the risk of metabolic syndrome (MetS) in Tunisian women. *Methods*. We analyzed a total of 2680 women aged between 35 and 70 years. Blood pressure, anthropometric indices, fasting glucose, and lipid profile were measured. The MetS was assessed by the modified NCEP-ATPIII definition. *Results*. The mean values of waist circumference, blood pressure, plasma lipids, and fasting glucose were significantly higher in postmenopausal than in premenopausal women, a difference that was no longer present when adjusting for age. Except for hypertriglyceridaemia, the frequency of central obesity, hyperglycemia, high blood pressure, and high total cholesterol was significantly higher in postmenopausal than in premenopausal women. After adjusting for age, the significance persisted only for hyperglycemia. The overall prevalence of MetS was 35.9%, higher in postmenopausal (45.7% versus 25.6%) than in premenopausal women. A binary logistic regression analysis showed that menopause was independently associated with MetS (OR = 1.41, 95% CI 1.10–1.82) after adjusting for age, residence area, marital status, family history of cardiovascular disease, education level, and occupation. *Conclusions*. The present study provides evidence that the MetS is highly prevalent in this group of women. Menopause can be a predictor of MetS independent of age in Tunisian women.

## 1. Introduction

The metabolic syndrome (MetS) has been identified as a constellation of metabolic and nonmetabolic disorders related to defects in insulin sensitivity that lead to a high risk for the development of type 2 diabetes and cardiovascular disease (CVD) [1]. The MetS is considered a problem of world proportions affecting both developed and underdeveloped countries in a rapidly progressive way [2–4]. The prevalence in different countries, nevertheless, varies considerably. Differences in genetic profile, eating habits, level of physical activity, age, gender, and lifestyle influence the prevalence of MetS and its components [5]. The prevalence of MetS varies also according to the characteristics of the population studied and the diagnostic criteria adopted [6]. It has been suggested that menopause is a contributing factor for this change in prevalence [7]. Menopause with its incidental hormonal changes appears to increase the risk of CVD independently of normal aging, and premenopausal women may be protected against CVD compared with men and postmenopausal women of a similar age [8]. Estrogen deficiency appears to be associated with an increased risk for the development of most of the clinical features comprising MetS [9]. Whether menopause has a casual effect on MetS is unclear. Few studies have investigated the effect of menopause on the development of the MetS independent of age [7, 10–12]. As there is no Tunisian report available concerning this subject, we undertook the present study to determine the prevalence of MetS and its components in pre- and postmenopausal women, as well as the association between the menopausal status and MetS.

## 2. Methods

### 2.1. Sampling

A total of 8007 individuals (mean age ± SD, 49.6 ± 9.7 years, range: 35–74 years; 3417 men (49.6 ± 9.8 years), 4590 women (49.5 ± 9.6 years)) were enrolled from a national Tunisian survey conducted from April 2004 to September 2005. The subjects were selected using two-stage stratified cluster sampling of households. The sampling frame was derived by the Tunisian National Institute of Statistics from the database of the 2004 census of the population; in each stratum 47 census districts were selected, with a probability proportional to size in number of eligible households (i.e., featuring at least one 35–74 years old household resident). At the second stage, 25 eligible households were sampled in each district; then for each household, all household residents from the targeted age range were to be included during the implementation of the field survey. This survey was integrated in a collaborative project funded by the European Commission to study epidemiological Transition and Health Impact in North Africa (TAHINA). The overall response rate was 79.5%; rates were generally lower in urban than rural areas and in men than in women. The study protocol was carried out according to the Declaration of Helsinki and has been ethically approved by the Tunisian Ministry of Health and the Tunisian National Council of Statistics (visa n°5/2005). All participants gave their free informed consent, after being thoroughly informed on the purpose, requirement, and procedures of the survey.

Among the 4590 women, participants who had one or more missing values of the risk factors necessary for a diagnosis of MetS were excluded, leaving 2814 persons. We excluded also smoker subjects because of the limited size of this group (42) and in order to eliminate the possible implication of smoking in the relationship between menopause and MS. For the analysis of the effect of menopause, we excluded those who had missing data regarding menopause, including premature menopause induced by surgery, chemotherapy, or radiation (62), and those who currently used hormone replacement therapy (HRT) (30). These exclusions were done in order to homogenize data for all participants. Ultimately, a total of 2680 (58%) women, aged 35–74 years, were included in the present study. They were divided in two groups: premenopausal and postmenopausal women. Menopause is defined as amenorrhea for the 12 months following the final menstrual period [13]. In our study postmenopausal women were defined as women with natural menopause whose current age was ≥1 year than their age of menopause and who did not receive HRT.

### 2.2. Data Collection

During the course of the interview, trained research nurses had to complete a questionnaire that contained data on sociodemographic characteristics including age, residence area, marital status, education level, occupation, smoking, and family medical history of CVD. A family history of CVD was defined as having at least one parent or sibling diagnosed with hypertension, stroke, or coronary heart disease. The age and the time elapses after menopause were included in the questionnaire. Marital status was categorized as single, married, widowed, or divorced. Education was classified into four categories: level 0: illiterate; level 1: low-level education (≤6 years of schooling); level 2: intermediate-level education (7–13 years of schooling); and level 3: higher education (≥14 years of schooling). Occupation was classified into two categories: employed and unemployed.

### 2.3. Measurements

Anthropometric measurements including weight and height were taken with the subjects' barefoot and wearing light clothing. Waist circumference (WC) was measured in the erect position at the middle between the tenth rib and the iliac crest. Body mass index (BMI) was calculated as the ratio of weight (kg) to the square of the height (m^2^). Systolic blood pressure (SBP) and diastolic blood pressure (DBP) were measured twice by mercury sphygmomanometer in the sitting position after they had been quietly seated for more than 15 min. The mean of the two measurements was used in the analyses. Blood samples were collected after a 12-hour overnight fast. Fasting plasma glucose (FPG) was determined by the glucose oxidase enzymatic method. Serum triglycerides (TG) were measured enzymatically after hydrolyzation of glycerol and total-cholesterol (TC) was measured by the cholesterol oxidase enzymatic method. All biochemical analyses were done using a Beckman reagent kit on a Beckman SYNCHRON CX7 analyzer.

### 2.4. Definition of the MetS

The National Cholesterol Education Program Adult Treatment Panel III (NCEP-ATPIII) clinical definition of the MetS requires the presence of three or more of the following criteria: WC ≥ 102 cm in men or ≥88 cm in women, TG ≥ 1.7 mmol/L, HDL-C < 1.03 mmol/L in men or <1.29 mmol/L in women, SBP ≥ 130 mmHg and/or DBP ≥ 85 mmHg, and FPG ≥ 6.1 mmol/L [14]. In the present study HDL-cholesterol (HDL-C) was not determined because at the time of the survey the method of dosage was the precipitation method, which was known for its large variability, and serum was not kept to measure it. Since TC has been shown to be directly related to mortality from coronary heart diseases, even in populations with low cholesterol concentrations [15], we used TC instead of HDL-C as previously reported [16–19]. The cut-off point used for TC was ≥5.2 mmol/L in men and women. Subjects who reported taking hypertension or diabetes medications were considered to have elevated BP or high FPG, respectively.

### 2.5. Statistical Analysis

Data are presented as mean (SD) for continuous variables and as number (percentage) for categorical variables. Differences in baseline characteristics between groups were analyzed by Student's* t*-test and *χ* ^2^ test for continuous variables and categorical variables, respectively. Associations of quantitative variables with menopausal status were examined by analysis of covariance (General Linear Model procedure) adjusting for age. The age-adjusted odds ratios (ORs) for risk factors for the MetS were calculated by logistic regression with age as a continuous variable. The relationship between the menopausal status and MetS was observed in a simple logistic regression model, with the OR and its confidence interval (CI) being estimated at 95%. Multivariate logistic regression analysis was used to assess the independent contribution of menopausal status to the presence of the MetS with an adjustment for age, residence area, marital status, family history of CVD, occupation, and education level. Data analysis was performed using the SPSS statistical package for Windows (version 15.0, SPSS, Chicago, IL, USA), and *P* value of less than 0.05 was considered significant.

## 3. Results

Anthropometric and biological characteristics of the study population stratified by menopausal status are presented in Table 1. The participants were between 35 and 74 years old, including 1311 (48.9%) premenopausal women with mean age 42.9 ± 5.0 years and 1369 (51.1%) postmenopausal women with mean age 57.5 ± 7.3 years. In addition to significantly higher age, postmenopausal women also had higher mean values of WC, SBP and DBP, and plasma TC, TG, and FPG compared to premenopausal women. After adjustment for age, the difference was no longer present.

The prevalence of the MetS and its components among all study participants and by menopausal status are summarized in Table 2. In all population, the most frequent component of MetS was high WC followed by high TG, high BP, high TC, and high FPG. Except for high TG, the prevalence of the other MetS components was significantly higher among postmenopausal women. However, when these factors were adjusted for age, the significance persisted only for high FPG. The overall prevalence of MetS was 35.9%. Prevalence of MetS amongst postmenopausal women was significantly higher than that in premenopausal women (45.7% versus 25.6%, *P* < 0.001).

Table 3 showed the ORs for MetS after adjustment for age groups, residence area, marital status, family history of CVD, education level, occupation, and menopausal status. The unadjusted and multivariate-adjusted ORs for the MetS in postmenopausal women were 2.45 and 1.41, respectively (95% CI 2.08–2.89 and 1.10–1.82, resp.) compared with premenopausal women. Multiple logistic regression analysis revealed that oldest age; urban area and widowed/divorced status were associated with increased ORs for the MetS. However, the multivariate model showed that family history of CVD, education level, and occupation were not associated with the MetS. In the multivariate analysis, when age was included as a continuous variable, the relationship between menopause and MetS remained statistically significant (OR = 1.42, 95% CI 1.10–1.84, *P* = 0.006).

Since the prevalence of MetS increases with age and due to the higher collinearity between age and menopausal status, we added a subgroup analysis on the women aged 45 to 54 for better clarifying the association between menopause and the occurrence of MetS. In this subgroup (*n* = 922), postmenopausal women (*n* = 521) had higher prevalence of MetS (42.8% versus 35.7%, *P* = 0.028) compared to premenopausal women (*n* = 401). In comparison to the premenopausal stage, the OR (95% CI) for MetS was 1.35 (1.03–1.76) for postmenopausal stage. In a multivariate model, adjusting for possible confounding factors, menopause was independently associated with MetS (OR = 1.39, 95% CI 1.05–1.84, *P* = 0.020), and this association was unchanged after adjusting for age. Urban residence was significantly associated with the occurrence of MetS in univariate and multivariate logistic regressions (Table 4).

## 4. Discussion

Today, CVD is one of the main causes of mortality of women in the world [20]. Various studies have shown that being affected by MetS increases the risk of CVD as well as morbidity of the disease [21, 22]. On the other hand, menopause with its incidental hormonal changes appears to increase the risk of CVD independently of normal aging, and premenopausal women may be protected against CVD compared with men and postmenopausal women of a similar age [8]. The relationship between menopause and MetS is controversial [8]. Rarer still are those that associate MetS with menopause. This lack of data, plus the importance of MetS as a cardiovascular risk factor, encouraged us to conduct this study, which was intended to determine the prevalence of MetS and its components in premenopausal and postmenopausal women, as well as the association between the menopausal status and MetS.

The overall prevalence of MetS in this study was 35.9%. Such data are consistent with those reported in a recent study carried out in the Tunisian population [3]. To the best of our knowledge, our study is the first to demonstrate the effect of menopause on the MetS in a representative population of Tunisian women. We found that the prevalence of MetS was significantly higher among postmenopausal women (45.7%) than among premenopausal women (25.6%). There is current controversy on whether MetS worsens with age alone or as a result of menopausal transition. In the present study, the prevalence of MetS increased with age. This finding was statistically significant in both univariate and multivariate analyses. In addition to the significant effect of age on the risk of MetS, the multivariate model showed that urban area was significantly associated with an increased risk of the MetS. Our findings showed also that the risk of MetS increased among widowed and divorced women compared to married women and that could be explained by the fact that married women are more likely to engage in positive health behaviors than widowed and divorced women [23].

With respect to menopausal status, a binary logistic regression analysis showed that postmenopausal stage was associated with an increased risk of the MetS adjusting for confounding variables such as age, residence area, marital status, family history of CVD, education level, and occupation.

To clarify the relationship between menopausal status and MetS, and to minimize the high collinearity between age and menopause, we focused our analyses in a smaller sample of women who are in similar age range. We analysed the subgroup of women aged 45–54 years. We found that the prevalence of MetS remained significantly increased in postmenopausal women and that the risk of MetS increased with postmenopausal stage, even after adjusting for possible confounding factors. These results are consistent with Korean, US, and Iranian studies [7, 10–12]. In contrast, other studies reported that postmenopausal women had higher prevalence of the MetS than premenopausal women, but this difference disappeared when adjusted for age [24–27]; the authors mentioned that the main risk factor for the increase in the prevalence of MetS was age [26].

Our finding adds to the growing body of evidence implicating menopause as a predictor of MetS independent of age. The mechanisms underlying the association between menopausal transition and MetS have not fully been elucidated. The decline in ovarian function is considered the main cause of this phenomenon [9]. A nine-year cohort study in various ethnic groups demonstrated that 13.7% of women were affected by MetS in menopause period and all components of MetS changed significantly in the studied women after menopause. This study demonstrated that the occurrence of MetS in postmenopausal women was the result of testosterone hormone secretion and change in all components of MetS [11]. It has been recently reported that menopause is closely related to insulin resistance and cardiovascular risk factors [12].

Although it is commonly believed that menopause is associated with weight gain, most studies do not reveal increases in BMI independent of normal aging [7, 12]. However, even in the absence of weight gain, body fat distribution changes across the menopause. Cross-sectional [28] and longitudinal studies [11] have shown that the menopausal transition is associated with a preferential increase in abdominal adiposity, independent of the effect of age and total body adiposity. In the present study we failed to detect an association between anthropometric measurements including BMI and WC and menopause result similar to that found in other studies [12, 26, 29]. The influence of menopause on BP is difficult to evaluate because menopause coincides with aging [30]. Some studies have reported a strong association between BP and menopause, but other studies have not [30, 31]. We found that mean SBP and DBP did not differ significantly between pre- and postmenopausal women after adjusting for age.

Menopause was found to be associated with an increase in TC and TG levels [32, 33] and a decrease in HDL-C [8, 34]. However, not all studies [8, 12, 29, 35] agree with this conclusion. In our study, variation in plasma lipid profile during menopause transition was age dependent. Increase in FPG in postmenopausal women has been reported [36], whereas other studies reported no significant changes in FPG levels between pre- and postmenopausal women [7, 29]; these findings are in accordance with our result.

Change in the rate of MetS components during menopausal transition is controversial [7, 12, 29]. Our findings showed no significant difference in the rate of MetS components across menopausal status adjusting for age except of hyperglycemia. It may be partly due to aging, but this alone cannot explain the rapid acceleration in the rate of MetS suffered by women during menopause [9]. In our population, the risk of MetS increased in postmenopausal period, even excluding the effect of age and other confounding factors. The absence of significant difference in incidence of MetS components except one of them between the two menopausal stages suggests that besides the aforementioned factors, other effective factors such as genetic or environmental ones contribute to the pathogenesis of the disease, which shows their effect in various ethnic groups differently.

Our study has strengths and limitations. The strengths include the large sample consisting of both urban and rural populations, a sound representation of the national population, and detailed information on potential confounding factors. As a cross-sectional study, the present analysis, however, is limited in its ability to elucidate a causal relationship. Another limitation was the absence of the measurement of HDL-C as explained above. Additionally, selection bias might have occurred during the exclusion of ineligible women.

## 5. Conclusion

The present study showed a high prevalence of MetS amongst women above 35 years of age. The MetS was more prevalent amongst postmenopausal women and the significance persisted when adjusted for age and other confounding factors. Although there was an independent association between menopausal status and the prevalence of MetS, further prospective studies are needed to confirm menopause as a risk factor for developing MetS.

## Figures and Tables

**Table 1:** Differences in anthropometric and biological parameters between pre- and postmenopausal women.

Variables	Premenopausal	Postmenopausal	*P* value
	Women (*n* = 1311)	Women (*n* = 1369)	
Age (years)	42.9 (5.0)	57.5 (7.3)	<0.001
BMI (kg/m²)	29.4 (9.3)	28.9 (10.5)	0.253
WC (cm)	91.7 (13.1)	93.7 (13.3)	<0.001
SBP (mmHg)	123.7 (17.2)	136.5 (22.1)	<0.001
DBP (mmHg)	74.4 (11.0)	80.5 (12.8)	<0.001
FPG (mmol/L)	4.71 (1.41)	5.30 (2.09)	<0.001
TC (mmol/L)	4.41 (0.98)	4.66 (1.03)	<0.001
TG (mmol/L)	1.94 (1.05)	2.02 (1.12)	0.048

Data are presented as mean (SD) and were compared by *t*-test. BMI: body mass index; WC: waist circumference; SBP: systolic blood pressure; DBP: diastolic blood pressure; FPG: fasting plasma glucose; TC: total cholesterol; TG: triglycerides.

**Table 2:** Prevalence of the metabolic syndrome and its components among all study participants and by menopausal status.

	All subjects *n* (%)	Premenopausal *n* (%)	Postmenopausal *n* (%)	*P* value
MetS components				
High WC	1768 (66.0)	830 (63.3)	938 (68.5)	0.004
High BP	1359 (50.7)	473 (36.1)	886 (64.7)	<0.001
High FPG	345 (12.9)	103 (7.9)	242 (17.7)	<0.001
High TC	599 (22.4)	221 (16.9)	378 (27.6)	<0.001
High TG	1410 (52.6)	673 (51.3)	737 (53.8)	0.195
MetS	961 (35.9)	335 (25.6)	626 (45.7)	<0.001

WC: waist circumference; BP: blood pressure; FPG: fasting plasma glucose; TC: total cholesterol; TG: triglycerides.

**Table 3:** Unadjusted and adjusted odds ratio for the metabolic syndrome as dependent variable and the associated factors as independent variables among all study participants.

	*n* (% MetS)	Unadjusted^a^ OR (95% CI)	*P* value^a^	Adjusted^b^ OR (95% CI)	*P* value^b^
Age (years)					
35–44	930 (21.0)	1		1	
45–54	922 (39.7)	2.48 (2.02–3.04)	<0.001	2.06 (1.60–2.65)	<0.001
55–64	532 (46.8)	3.31 (2.62–4.18)	<0.001	2.39 (1.70–3.37)	<0.001
65–74	296 (51.0)	3.92 (2.97–5.17)	<0.001	2.88 (1.96–4.24)	<0.001
Residence area					
Rural	**1130 (29.2)**	1		1	
Urban	**1550 (40.7)**	**1.66 (1.41–1.96)**	**<0.001**	**1.79 (1.49–2.14)**	**<0.001**
Marital status					
Married	**2205 (33.8)**	1		1	
Single	61 (37.7)	1.18 (0.70–2.00)	0.524	1.25 (0.71–2.19)	0.428
Widowed/divorced	**414 (46.6)**	**1.71 (1.38–2.11)**	**<0.001**	**1.26 (1.00–1.59)**	**0.043**
Family history of CVD					
No	2592 (35.4)	1		1	
Yes	75 (52.0)	1.97 (1.24–3. 13)	0.004	1.39 (0.85–2.28)	0.178
Education level					
Illiterate	1527 (37.7)	1		1	
Lower	804 (35.3)	0.90 (0.75–1.07)	0.254	1.21 (0.98–1.49)	0.077
Medium	282 (29.8)	0.70 (0.53–0.92)	0.011	0.91 (0.66–1.25)	0.584
Higher	60 (23.3)	0.50 (0.27–0.92)	0.026	0.74 (0.37–1.48)	0.405
Occupation					
Employed	322 (30.1)	1		1	
Unemployed	2287 (37.0)	1.36 (1.06–1.75)	0.016	0.86 (0.64–1.15)	0.312
Menopause status					
Premenopausal	**1311 (25.6)**	1		1	
Postmenopausal	**1369 (45.7)**	**2.45 (2.08–2.89)**	**<0.001**	**1.41 (1.10–1.82)**	**0.007**

OR: odds ratio; CI: confidence interval; MetS: metabolic syndrome; CVD: cardiovascular disease.

^a^Univariate logistic regression.

^b^Multivariate logistic regression analysis including MetS as the dependent variable and age groups, residence area, marital status, family history of CVD, educational level, occupation, and menopause status as independent variables.

**Table 4:** Unadjusted and adjusted odds ratio for the metabolic syndrome as dependent variable and the associated factors as independent variables among participants aged 45 to 54 years.

	*n* (% MetS)	Unadjusted^a^ OR (95% CI)	*P*value^a^	Adjusted^b^ OR (95% CI)	*P*value^b^
Residence area					
Rural	**384 (34.6)**	1		1	
Urban	**538 (43.3)**	**1.44 (1.10–1.89)**	**0.008**	**1.49 (1.11–1.99)**	**0.007**
Marital status					
Married	767 (39.1)	1		1	
Single	15 (33.3)	0.77 (0.26–2.29)	0.650	0.61 (0.19–2.01)	0.427
Widowed/divorced	140 (43.6)	1.20 (0.83–1.73)	0.322	1.16 (0.79–1.71)	0.422
Family history of CVD					
No	20 (50.0)	1		1	
Yes	898 (39.5)	1.53 (0.63–3.71)	0.348	1.42 (0.56–3.58)	0.452
Education level					
Illiterate	485 (38.1)	1		1	
Lower	326 (42.0)	1.17 (0.88–1.56)	0.268	1.14 (0.84–1.55)	0.391
Medium	94 (38.3)	1.00 (0.63–1.58)	0.978	0.95 (0.58–1.55)	0.845
Higher	15 (40.0)	1.08 (0.37–3.08)	0.884	1.11 (0.36–3.38)	0.845
Occupation					
Employed	123 (35.8)	1		1	
Unemployed	770 (41.0)	1.25 (0.84–1.85)	0.270	1.36 (0.89–2.10)	0.151
Menopause status					
Premenopausal	**401 (35.7)**	1		1	
Postmenopausal	**521 (42.8)**	**1.35 (1.03–1.76)**	**0.028**	**1.39 (1.05–1.84)**	**0.020**

OR: odds ratio; CI: confidence interval; MetS: metabolic syndrome; CVD: cardiovascular disease.

^a^Univariate logistic regression.

^b^Multivariate logistic regression analysis including MetS as the dependent variable and residence area, marital status, family history of CVD, educational level, occupation, and menopause status as independent variables.
